# An accurately supervised motion-aware deep network for non-contact pain assessment of trigeminal neuralgia mouse model

**DOI:** 10.22514/jofph.2024.008

**Published:** 2024-03-12

**Authors:** Zhiheng Feng, Mingcai Chen, Jue Zhang, Xin Peng

**Affiliations:** ^1^Academy for Advanced Interdisciplinary Studies, Peking University, 100871 Beijing, China; ^2^State Key Laboratory for Novel Software Technology, Nanjing University, 210023 Nanjing, Jiangsu, China; ^3^College of Engineering, Peking University, 100871 Beijing, China; ^4^Department of Oral and Maxillofacial Surgery, Peking University School of Stomatology, 100081 Beijing, China

**Keywords:** Oral diseases, Trigeminal neuralgia, Pain assessment, Convolutional neural networks

## Abstract

Pain assessment in trigeminal neuralgia (TN) mouse models is essential for exploring its pathophysiology and developing effective analgesics. However, pain assessment methods for TN mouse models have not been widely studied, resulting in a critical gap in our understanding of TN. With the rapid advancement of deep learning, numerous pain assessment methods based on deep learning have emerged. Nonetheless, these methods have some limitations: (1) insufficiently objective supervision signals for training, (2) failure to account for the dynamic behavioral characteristics of mouse models in the constructed models and (3) inadequate generalization ability of the models. In this study, we initially constructed an objective pain grading dataset as the ground truth for model training, which remedy the limitations of prior studies that relied on subjective evaluation as supervisory signals. Then we proposed a novel deep neural network, named trigeminal neuralgia pain assessment network (TNPAN), which fuses the static texture characteristics and dynamic behavioral characteristics of mouse facial expressions. The promising experimental results demonstrate that TNPAN exhibits exceptional accuracy and generalization capability in pain assessment.

## 1. Introduction

Trigeminal neuralgia (TN) is a debilitating condition afflicts a substantial portion of the global population. Patients with TN experience excruciating, knife-like, sharp and lightning-like pain emanating from the trigeminal nerve [1], which imparts a significant burden. Despite its prevalence, the pathophysiological basis of TN remains inadequately understood [2]. Mouse models of TN have been developed to explore the underlying pathophysiology and potential therapies for this condition. Nevertheless, the development of a reliable pain assessment method for these models remains a significant challenge. The current lack of widely adopted pain assessment for TN mouse models has impeded the advancement of research in this area. Thus, there is a pressing need to develop and validate more robust pain assessment tools to quantify pain intensity in these models precisely.

The current pain assessment methods for mice can be classified into stimulus-evoked and non-stimulus-evoked. Stimulus-evoked methods involve applying mechanical, thermal (heat or cold), or chemical stimuli that alter the mice’s behavior, followed by observing their responses. Non-stimulus-evoked methods measure spontaneous behaviors such as burrowing, nesting, weight-bearing or facial grimace scale. The facial grimace scale is extensively utilized as a pain assessment method for animals, given that facial expressions can be predominantly considered a reflexive response to noxious stimuli in animal models, which confers a natural edge in appraising pain intensity. For example, Langford *et al*. [3] proposed the Mouse Grimace Scale (MGS) by leveraging the facial expressions of experimental mice. Dalla Costa *et al*. [4] introduced the Horse Grimace Scale (HGS) for pain assessment by analyzing horses’ facial expressions. The facial grimace scale generally includes various facial action units (AUs), such as the eyes, ears, nose, cheeks and other facial regions. However, the application of the facial grimace scale for pain assessment is hindered by the time-consuming and the introduction of subjective elements. Even a seasoned pain assessment expert must dedicate multiple hours to scrutinize a mouse’s facial expressions, making the evaluation process taxing [5]. The existence of subjective factors among evaluators can considerably contribute to the inter-observer variability in pain assessment. These factors significantly elevate the complexity of pain assessment.

In recent years, with the rapid development of deep learning, many non-contact pain assessment models have emerged that can be applied to humans [6, 7, 8]. Motivated by these models, numerous researchers have initiated their application and dissemination on mice. Non-contact pain assessment models can be classified into two main categories: one utilizes computer vision techniques to quantify a series of facial features of experimental animals, such as eye socket position, nose size, and ear position, and uses these quantitative results to perform pain assessment. The other category employs supervised machine learning methods to train a pain assessment model, which is then used for pain assessment. While the previous research has propelled the field of pain assessment, some common issues exist: (1) The supervisory signals utilized in machine learning training lack objective standards. The ground truth (GT) for pain grading frequently stems from annotators’ subjective evaluations, leading to biased models. (2) Prior models typically emphasize the static structural features of experimental mice, disregarding the dynamic behavioral features that are vital for pain assessment. (3) The models’ ability to generalize is limited. For example, Tuttle *et al*. [9], and Vidal *et al*. [10] trained their models on white laboratory mice, limiting the applicability of their models to only white laboratory mice.

This paper presents a non-contact trigeminal neuralgia pain assessment network (TNPAN) based on dual-channel feature fusion to address the issues above. The TNPAN model possesses three distinct advantages: (1) objective supervisory signals, by virtue of employing pain grading ground truth based on physiological signals of mice, rather than the subjective judgments of researchers in the dataset; (2) it incorporates both static texture characteristics and dynamic behavioral characteristics of the TN mouse model; and (3) it demonstrates a robust generalization ability. TNPAN consists of two components: a motion awareness network (MAN) and a 3D convolutional neural network (3D-CNN). In MAN, we incorporated the consideration of behavioral features of mouse model, namely, the pupil and forelimb movements. Mice exhibit significant changes in pupil size and tend to alleviate pain by rubbing their face with their forelimbs when experiencing pain. These pain-related behavioral features have been overlooked in previous models. In 3D-CNN, we employed 3D convolutional kernels to capture texture features that are related to pain in the TN mouse model. This is because facial texture changes occur when mice experience pain. Finally, MAN and 3D-CNN are integrated into parallel to compose TNPAN. To assess the performance of TNPAN, we conducted a comparison with the state-of-the-art benchmark models. The experimental results demonstrated that TNPAN outperformed the existing models in accurately assessing pain in TN mice. To evaluate the generalization capability of TNPAN, we applied it to the mouse model of infraorbital nerve constriction injury (IoN-CCI). The results indicated that TNPAN demonstrated strong generalization capability in the pain assessment of mice, as the predicted pain grades were consistent with those obtained through the von Frey filament test [11]. The contributions of this work are:

1. We constructed an objective pain grading dataset for model training, which remedied the limitations of prior studies that relied on subjective evaluation as supervisory signals.

2. We propose a deep neural network named TNPAN, which takes into account both the static texture characteristics and dynamic behavioral characteristics of the TN mouse model, achieving higher accuracy in pain assessment. This dual-channel feature fusion strategy provides directions for the future development of pain assessment models.

3. TNPAN demonstrates robust generalization, as it can not only be applied to acute TN mouse models but also for mice with infraorbital nerve ligation (IoN-CCI) injury.

## 2. Related work

### 2.1 Pain assessment in animals

Pain assessment is essential for pain management and selection of appropriate therapeutic interventions. Facial action units and landmarks have been utilized to study emotional status in individuals. McLennan *et al*. [12] developed a system incorporating these tools to extract features from specific regions of the face, including the eyes, nose and mouth, using the histogram of oriented gradients (HOG) technique. The progress of computer vision algorithms in detecting human facial expressions has led to recent research exploring the application of similar tools to study animal behaviors. Langford *et al*. [3] introduced the mouse grimace scale (MGS), a facial action coding system for mice. The MGS relies on five facial features to assess pain: orbital tightening, nose bulge, cheek bulge, ear position and whisker change.

However, in using these methods, researchers are faced with the challenge of identifying the frontal faces of animals from the video, which is a time-consuming task. To eliminate the need for manual image extraction, the Rodent Face Finder software was developed to automatically identify and extract video frames of mice facing the camera [13]. Nevertheless, this software is still subject to the influence of annotator bias. Furthermore, as animals cannot self-report their pain status, progress on automatic pain assessment based on facial expression remains limited [14].

### 2.2 Relation to prior work

The studies by Kopaczka *et al*. [15], Tuttle *et al*. [9], and Vidal *et al*. [10] are the most relevant to our research. Kopaczka *et al*. [15] proposed a deep learning-based method using convolutional neural networks (CNNs) to detect the eye region for black mice. They recorded the mice against a red background to simplify the task. Tuttle *et al*. [9] developed an end-to-end framework based on the Inception V3 architecture [16] for the binary classification of pain in white-furred mice (*i.e.*, pain versus no pain), using deep learning techniques. Vidal *et al*. [10] employed the You Only Look Once (YOLO) framework to detect the mice’s face and proposed a novel CNN for eye region extraction and grimace pain prediction. However, there are common limitations between their study and those of Kopaczka *et al*. [15] and Tuttle *et al*. [9] Firstly, the ground truth used in their training process is of low quality due to the subjective factors involved in pain grading. Secondly, the model proposed by Tuttle and Vidal is only applicable to white-furred mice, while most mice used in pain research have black fur. Thirdly, their methods focus only on the structural features of the mice’s faces while ignoring the dynamic behavioral characteristics related to pain. In our study, we propose a framework for pain assessment that takes into account these factors.

## 3. Materials and methods

### 3.1 Strains of mice and compounds

All mice used in this study were wild-type (C57BL/6J), weighing between 30 g and 50 g and aged 2–5 weeks. They were obtained from the Model Animal Research Center of Nanjing University (Nanjing, China) and bred in Nanjing Brain Observatory Laboratory Animal Centre (NBO) with a specific pathogen-free (SPF) environment. The mice were housed under a 12 h light, 12 h dark cycle (lights on at 08:00 h) in a temperature-controlled environment (22 ± 1 ℃) with ad lib access to food and tap water. Sodium chloride was obtained from Shanghai Aladdin Biochemical Technology Co. Cal-520 AM was generously provided by Nanjing Brain Observatory.

### 3.2 Data source

This section presents a detailed description of the experimental procedures and data acquisition (as depicted in Fig. 1). Objective pain grading was performed in the TN mouse model through the infraorbital nerve activity assay and the mouse facial pain threshold assay.

To achieve an objective pain grading in the TN mouse model, the MGS method previously used by others was avoided as it introduces the annotator’s subjective factors. Instead, this study focuses on the activity of the IoN in the trigeminal nerve signaling circuit and the facial pain threshold as physiological indicators related to mice pain, which have objective bases for reflecting the pain intensity of mice [17].

**Fig. 1.** The illustration of the data acquisition procedures. Two experiments were conducted to objectively assess pain intensity in the TN mouse model: the infraorbital nerve activity assay and the mouse facial pain threshold assay. (a) Thirty-six mice were divided into the IoN activity assay group (n = 18) and the facial pain threshold assay group (n = 18). The IoN activity was measured by fluorescence microscopy in the first group, while the facial pain threshold was measured using von Frey filaments in the second group. The results of these assays were used to establish objective pain grading criteria (ground truth). (b) The facial expressions of 18 mice exposed to varying concentrations of saline are recorded using a video camera. A deep learning model (TNPAN) proposed by this study is trained in a supervised manner for non-contact pain assessment.

#### 3.2.1 Infraorbital nerve activity assay

Initially, we randomly allocated the mice into six groups, with three mice in each group. Our approach to random grouping was as follows: assigning a unique number to each of the 18 mice and subsequently utilizing the random number generator in Excel to allocate the mice into six groups in a randomized manner. The mice were then placed into an anesthesia box, and gas anesthesia was administered, with an induction dose of 2–5 levels and a gas flow of 1–1.5 L/min. Anesthesia was assessed based on the breathing of the mice. The scalp was shaved, and the skin was prepared along the right gingival buccal margin, at the level of the first molar, and a longitudinal incision of approximately 1 cm was made towards the nose and mouth to expose the infraorbital nerve (IoN) and surrounding tissue. Next, 0.5 µL of Cal-520 AM calcium-sensitive dye was aspirated using a microinjector. The microinjector was secured on the brain stereotaxic instrument, inserted into the designated site of the infraorbital nerve, and left for a while before the injection was performed using the microinjection pump.

After completion, the needle was left in place for approximately 5–10 minutes before being withdrawn at a constant speed. The room temperature was maintained at approximately 28 ℃ throughout the procedure, and sufficient food and water were provided before and after the surgery. All procedures were carried out under sterile conditions. Half an hour following awakening from anesthesia, fluorescence imaging was conducted on the infraorbital nerves of the mice using imaging software (GINKGO MTPM V1.0, Transcend Vivoscope Biotech Co., Ltd, Beijing, China). The imaging parameters were adjusted to ensure clear visualization of the IoN in the visual field, as shown in Fig. 2. The mice (n = 18, 9 male and 9 female) were divided into six groups: 0.9% group, 6% group, 12% group, 18% group, 25% group and 30% group. During the experiment, the corresponding concentration of NaCl (Sodium Chloride) solution was dropped onto the cornea of the mice, and infraorbital nerve activity was recorded on video at 10 Hz simultaneously. Throughout the experimental procedure, recording the mice’s body weight was essential. Mice exhibiting weight fluctuations exceeding 10% were excluded from the experiment. Furthermore, mice displaying adverse anesthetic reactions or surgical complications were also subject to exclusion.

**Fig. 2.** The activity of IoN assays. (a) Result of IoN exposure. (b) The typical IoN result of fluorescent staining. Scale bars, 5 mm. IoN: infraorbital nerve; F_ROI_: the fluorescence intensity of the region of interest.

The activity of IoN (Δ*F/F*) was calculated from videos by the following formula : (1) *F_ROI_ = F_raw_ – F_b_*, where *F_ROI_* is the intensity trace subtracted from the video background, *F_raw_* is the average intensity curve on the ROI (region of interest) of IoN body, *F_b_* is the baseline fluorescence of the background, by the minimum value of the Mosaic image stack; (2)*F_con_ = F_ring_ – F_b_*, where *F_con_* for z axis pollution intensity, *F_ring_* for IoN ring area around the original strength; (3) *F_sig_ = F_ROI_ – α × F_con_*, where *F_sig_* represents the actual activity of IoN after subtracting the background drift and Z-axis pollution, and α represents the pollution level, α = 0.9 in this paper; (4) Δ*F/F = F_sig_/F*_0_, where *F*_0_ is the baseline values for *F_ROI_*, *F_ROI_* average estimate. Assumptions of normality were tested with the D’Agostino-Pearson normality test. All 2-group comparisons were calculated using Student’s *t*-test (two-tailed, paired or unpaired), when data followed a Gaussian distribution.

#### 3.2.2 Facial pain threshold assay

We randomly selected another 18 mice and divided them into six groups of three mice each. The mice were then subjected to gas anesthesia in an anesthesia box, with an induction anesthesia dose of 2–5 levels and a gas flow of 1–1.5 L/min. After the mice were anesthetized, they were immobilized on a brain stereotaxic apparatus. The scalp of the mice was depilated, and the skin was prepared and sterilized. A custom metal head post was fixed onto the mouse’s head using dental cement adhesion. The room temperature was maintained at around 28 ℃ during the entire operation, and sufficient food and water were provided before and after the operation.

All procedures were carried out under sterile conditions. Following a recovery period of one week after surgery, we measured the facial pain threshold of mice using von Frey filaments (0.002–1.4 g, Shanghai Yuyan Instruments Co., Ltd). A series of von Frey filaments consisting of eight filaments (0.02 g, 0.04 g, 0.07 g, 0.16 g, 0.4 g, 0.6 g, 1.0 g and 1.4 g) are selected. Starting from the 0.02 g filament, the Up-and-Down method [14, 18, 19, 20] is used to stimulate the mouse’s face. The filament should be bent into a “C” or “S” shape and applied for 6–8 seconds. The mouse’s withdrawal response is then observed and recorded: a negative response “O” is recorded if there is no response from the mouse, and a positive response “X” is recorded if there is a flinching or resistance response. In the event of a negative response, the next larger von Frey filament is used to stimulate the face. If there is a positive response, the next smaller filament is applied, with a few seconds between each stimulation. This process is repeated until “OX” or “XO” responses are observed. To obtain a sequence of responses consisting of combinations of “O” or “X”, four additional tests are conducted after the first “OX” or “XO” response. The filament size used in this sequence is recorded as the facial pain threshold of the mouse’s face. After a one-minute interval, we stimulated the right cornea of each mouse with a specific concentration (0.9%, 6%, 12%, 18%, 25%, 30%) of normal saline and then repeated the facial pain threshold measurement using the same protocol as before.

#### 3.2.3 Video capture

First, 18 mice were sequentially placed into an anesthesia box and subjected to gas anesthesia. The induction dose was 2–5, and the gas flow rate was 1–1.5 L/min. The level of anesthesia in each mouse was determined based on its respiratory condition. After anesthesia, the mice were fixed on a stereotactic apparatus and a metal headpiece was mounted on their head. The scalp of the mouse was shaved and sterilized, and then the scalp was cut to expose the skull. The customized head post was affixed to the mouse’s head using dental cement. The entire surgical procedure was carried out at a room temperature of around 28 ℃, and the mice were provided with sufficient food and water before and after surgery. All procedures were performed under sterile conditions. After surgery, the mice were allowed to recover for one week to eliminate any potential influence of surgery on the acute trigeminal neuralgia experiment. The experiment was conducted after the recovery period.

Before the experiment, an infrared camera (CY-UB300, 2048 × 1536 pixels, 30 Hz frame rate) was positioned beside the left facial area of the mouse, with the jaw facing 90° away from the camera direction, and a distance of approximately 5 cm between them, as shown in Fig. 3. The mice were allowed to adapt to the experimental environment for 10 minutes. The next step involved dividing the 18 mice into six groups, and each group was subjected to the instillation of specific concentrations (0.9%, 6%, 12%, 18%, 25%, 30%) of saline solution (2 mL each time) on the right cornea to simulate acute TN. After the saline solution was applied, facial videos of the TN mice were recorded for 10 minutes. After the experiment, the mouse cornea was washed with water to avoid irreversible damage caused by high-concentration salt water.

**Fig. 3.** The experimental apparatus for recording facial expressions of TN mouse model. NaCl: sodium chloride.

### 3.3 Image pre-processing pipeline and human annotation

Although the heads of the mice were immobilized in this experiment, there were still slight movements of the face during imaging, and there were differences in the background among different mice. To eliminate the influence of the factors mentioned above, this study cropped the images to only include the facial region of the mouse, and uniformly resized the cropped images to a size of 512 × 512 pixels. In addition, we removed excessively blurry images caused by mouse movement to improve the overall image quality of the dataset. After the above pre-processing pipeline, 9882 images are available for NaCl 0.9% group, 9516 images are available for NaCl 6% group, 10,437 images are available for NaCl 12% group, 6273 images are available for NaCl 18% group, 9408 images are available for NaCl 25% group, 5544 images are available for NaCl 30% group. To balance the sample size for each pain level and reduce the cost of the video labeling. We selected 3000 images each from the no pain (NP) group, moderate pain (MP) group and high pain (HP) group.

To introduce the dynamic behavioral characteristics of the TN mouse model, this study invited five experienced experimenters to perform meticulous manual annotation of the pupil and forelimb key points of each mouse in the image, as shown in Fig. 4. We annotated a total of eight key points, with four marked at the upper (pupil1), lower (pupil2), left (pupil3), and right (pupil4) boundaries of the pupil. The other four key points were labeled at the front end (limb1), mid-front (limb2), mid-back (limb3), and rear end (limb4) of the forelimb.

**Fig. 4.** The annotation results of mouse pupils and forelimbs. (a) the original image of the mouse face. (b) yellow dots in the orange region indicate the annotation results of mouse pupils, and yellow dots in the blue region indicate the annotation results of mouse forelimbs.

Each image in the dataset is annotated with a pain grading label (NP, MP, HP) and the positions of eight key points related to pain behavior. The set of 9000 images with pain labels and mouse keypoint annotations constitutes an objective pain grading dataset.

### 3.4 Proposed trigeminal neuralgia pain assessment network

The proposed trigeminal neuralgia pain assessment network (TNPAN) consists of two parts, the motion awareness network (MAN) and the three-dimensional deep convolutional neural network (3D-CNN) as shown in Fig. 5. The MAN is employed to extract the dynamic behavioral characteristics of the TN mouse, providing identification results for the key points of the mouse’s pupils and forelimbs. The 3D-CNN is utilized to extract the static texture characteristics of the mouse’s face, specifically to provide the texture and structural features associated with pain. Finally, the features from the two channels of MAN and 3D-CNN are fused in a cascaded manner to provide the final pain assessment results.

**Fig. 5.** An overview of the trigeminal neuralgia pain assessment network (TNPAN), which consists of two neural networks: motion awareness network (MAN) and 3D-CNN. Each cube is a convolutional layer, and the black line is ReLU connections, while the arc shows the shortcut connection. The two networks are cascaded by a fully connected layer to evaluate the pain intensity with a fixed model reuse strategy. 3D-CNN: 3D convolutional neural network.

MAN is composed of a ResNet-50 [21] and eight deconvolutional layers. When ResNet was first proposed in 2015, it achieved first place in the ImageNet image classification task, and subsequently its excellent performance led to its widespread application in various object recognition tasks based on convolutional neural networks, such as ArtTrack [22], DeepCut [23], and DeepCut2 [24]. Therefore, in this study, we introduced ResNet-50 as a keypoint detector into MAN. The integrated framework is illustrated in Table 1. In order to output the detection results of the mouse’s pupil and key points of the forelimbs, we replaced the softmax layer after the “Conv 2d-5-x” convolutional layer in ResNet-50 with eight deconvolutional layers, with each deconvolutional layer outputting a score map corresponding to the key point. The values in the score map reflect the probability of the keypoint being located at a specific position [23, 24]. To enhance the robustness of MAN, during training, we labeled the area within 5 pixels of the key points as 1 and the area outside 5 pixels as 0. To improve the performance and speed up the convergence of MAN, in this study, the initialization parameters of ResNet-50 in MAN were derived from the pre-trained parameters on ImageNet.

**Table 1.** Deep learning architecture for motion awareness network.

Layer	Output shape	Activation	Kernel size	Stride
Conv 2d_1	256 × 256 × 64	ReLU	7 × 7, 64	2
Conv 2d_2_x	128 × 128 × 256	ReLU	$$\left[\begin{array}{cc} 1\times 1, & 64\\ 3\times 3, & 64\\ 1\times 1, & 256 \end{array}\right]\times 3$$	2
Conv 2d_3_x	64 × 64 × 512	ReLU	$$\left[\begin{array}{cc} 1\times 1, & 128\\ 3\times 3, & 128\\ 1\times 1, & 512 \end{array}\right]\times 4$$	2
Conv 2d_4_x	32 × 32 × 1024	ReLU	$$\left[\begin{array}{cc} 1\times 1, & 256\\ 3\times 3, & 256\\ 1\times 1, & 1024 \end{array}\right]\times 6$$	2
Conv 2d_5_x	32 × 32 × 2048	ReLU	$$\left[\begin{array}{cc} 1\times 1, & 512\\ 3\times 3, & 512\\ 1\times 1, & 2048 \end{array}\right]\times 3$$	1
Deconvolutional Layers	64 × 64 × 8	-	-	-
Readout Layers	2 × 8	-	-	-
Fully Connected Layer	16 × 1	-	-	-
Softmax Classifier	3 × 1	-	-	-

During training, stochastic gradient descent (SGD) [25] was utilized to minimize the cross-entropy loss function between the predicted results of MAN and the ground truth. In the experiments where MAN was used for pain assessment alone, we introduced eight readout layers after the deconvolution layers in MAN to read the coordinates of the eight key points of the mouse. The final activation layer was a softmax layer to ensure that the sum of the probabilities of the pain assessment results was one.

The 3D-CNN was primarily utilized to extract the static texture characteristics of the mouse facial region, comprising six 3D convolutional layers, three max-pooling layers, and one fully connected layer. The network architecture is illustrated in Table 2. Each 3D convolutional layer consists of several 2D convolutional kernels with time parameters. The 2D convolutional kernels with time parameters have a wider dynamic range, providing sufficient details for representing pain features. The pooling layers serve as downsampling layers, preserving the most important features while reducing the number of features and parameters in the model. The role of the fully connected layer is to condense the feature maps extracted by the preceding convolutional layers into a single vector. The feature map output by 3D-CNN is a tensor, with the input of *H × W × C* and output of *H × W × C'*.

**Table 2.** Deep learning architecture for 3D-CNN.

Layer	Output shape	Activation	Kernel size	Stride	Num of kernel
Conv 3d_1	16 × 512 × 512 × 32	ReLU	3 × 3 × 3	1 × 1 × 1	32
Conv 3d_2	16 × 512 × 512 × 32	ReLU	3 × 3 × 3	1 × 1 × 1	32
Max Pooling_1	8 × 256 × 256 × 32	-	-	2 × 2 × 2	-
Conv 3d_3	8 × 256 × 256 × 64	ReLU	3 × 3 × 3	1 × 1 × 1	64
Conv 3d_4	8 × 256 × 256 × 64	ReLU	3 × 3 × 3	1 × 1 × 1	64
Max Pooling_2	4 × 128 × 128 × 64	-	-	2 × 2 × 2	-
Conv 3d_5	4 × 128 × 128 × 128	ReLU	3 × 3 × 3	1 × 1 × 1	128
Conv 3d_6	4 × 128 × 128 × 128	ReLU	3 × 3 × 3	1 × 1 × 1	128
Max Pooling_3	2 × 64 × 64 × 128	-	-	2 × 2 × 2	-
Fully Connected Layer	16 × 1	-	-	-	-
Softmax Classifier	3 × 1	-	-	-	-

Where H and W represent the height and width of the feature map, and C and C’ denote the input and output channel numbers. The size of the 3D convolution kernel is *h × w × c*, where h and w indicate the height and width of the kernel, and c denotes the temporal depth. The output of each convolutional kernel can be represented as:

$$y=F\left(x|W,\text{\{}W_{d_{n}}{\text{\}}}_{n=1}^{N}\right)$$

Where W represents the weights of the 3D convolutional neural network (CNN) under fixed temporal parameters, while *W_d__n_^N^_n=1_* denotes a series of parameters in the 3D-CNN. It should be noted that the output of a convolutional neural network needs to be activated by the ReLU non-linear function. F represents all convolution operations and can be expressed as:

$$F\left(x\right)=\overset{N}{\underset{n=1}{⋃}}W_{d_{n}}\sigma \left(\text{Wx}\right)$$

Here, σ represents the ReLU non-linear function, and U represents the concatenation operation. In each convolutional module, the feature map *x* from the previous layer is convolved with the convolutional kernel, resulting in N new feature maps *S_1_, S_2_,..., S_N_*, where *S_n_ ϵ R^H×W×C_n_^*. Each intermediate convolutional layer has a fixed temporal parameter and spatial size. These intermediate feature maps *S_n_^N^_n=1_* are concatenated to form a simple tensor.

$$E=\frac{1}{K}\sum_{k=1}^{K}\;\text{|}\hat{o_{k}}-o_{k}\;{\text{|}}_{2}^{2}$$

Here, K denotes the length of a given sequence of images. The output of the final convolutional layer is flattened and connected to a fully connected layer of size 1 × 16. In experiments using 3D-CNN for pain assessment alone, the output layer had three dimensions representing three different pain intensities (0, 1, 2). The final activation layer is a softmax layer, ensuring that the sum of the probabilities of the pain assessment results is equal to 1. During the training of 3D-CNN, the cross-entropy loss function and SGD optimization algorithm are utilized. The initial learning rate is set to 0.0001, and the dataset is split into a training set and a test set with an 80% to 20% ratio. The epoch value is set to 100, and the batch size is set to 32. The training is performed end-to-end.

The final step is to cascade MAN and 3D-CNN using the fixed model reuse (FMR) strategy, which involves reusing the learned parameters of the 3D-CNN while only updating the parameters of MAN during the training process. During the concatenation process, two key considerations are applied: (1) fixed model reuse: since MAN is responsible for providing the key point recognition results of TN mice, the network weights of MAN are no longer updated during the training process of TNPAN. (2) Design of fully connected layer: since the deconvolution layer of MAN outputs a score-map, to concatenate the score-map with the features extracted by 3D-CNN, we added eight readout layers after MAN, which respectively output the coordinates of the eight key points of TN mice. These coordinates are flattened into a 1 × 16 vector and concatenated with the fully connected layer of 3D-CNN to form a 1 × 32 vector. The last layer of TNPAN is a softmax activation layer, which ensures that the sum of probabilities of pain evaluation results (0 for no pain, 1 for moderate pain, and 2 for high pain) equals to 1.

During the training of MAN, the dataset containing 9000 labeled images of mouse pupils and forelimb key points was used. The dataset was split into a training set (80%) and a test set (20%), and the evaluation metric for the MAN network was the error, which was defined as the pixel distance between the model’s predicted key points and the ground truth key points. After training the MAN network on a dataset of 9,000 annotated images of mice pupils and forelimb key points, the fixed model reuse (FMR) strategy was employed to cascade the MAN network with the 3D-CNN to form TNPAN. Then, the stochastic gradient descent (SGD) algorithm was used to optimize the network parameters of TNPAN, with only the parameters of the 3D-CNN and fully connected layers being adjusted. In the experiments of using MAN, 3D-CNN and TNPAN separately for pain assessment, the evaluation metrics of the models were accuracy (%), precision (%), recall (%), F1 score and area under the curve (AUC).

All training and evaluation were performed on a Windows 10 Professional server with an NVIDIA 1080Ti GPU (Graphics Processing Unit) and an AMD Ryzen 7 2700X Eight-Core Processor 3.70 GHz CPU (Central Processing Unit). The programming language used was Python 3.6 produced by the Python Software Foundation (PSF, which is registered in the United States), and the deep learning framework used was PyTorch 1.9. The training process was repeated three times, with an evaluation conducted after each training cycle. The final evaluation result is the average of the three evaluations.

### 3.5 Construction of the IoN-CCI mouse model and data collection

In machine learning, although model accuracy is undoubtedly essential, it is crucial not to overlook the model’s generalizability. To evaluate the generalizability of TNPAN, this study aimed to apply TNPAN to the mouse model of trigeminal neuralgia induced by IoN-CCI surgery. The precise experimental protocol is outlined as follows: (1) Six C57BL/6J mice (3 female and 3 male) with a body weight ranging between 30 g to 50 g and aged two weeks were included in the study, of which three mice underwent IoN-CCI surgery, while the remaining three mice underwent sham surgery. All enrolled mice were initially healthy. The strategy for random grouping was the same as described in section 3.3, resulting in the random allocation into Sham and IoN-CCI groups. (2) The standard procedure for IoN-CCI involves depilating the mouse and making a vertical incision, approximately 1 cm in length, along the right gingival cheek margin towards the nose. The IoN is exposed as shown in Fig. 6a, and the surrounding tissue is dissected, followed by the application of a 4.0 chromium wire to loosely ligate the nerve, with the tension reduced only enough to delay nerve conduction without completely blocking it, while still maintaining blood circulation. Finally, the incision is closed with a 4.0 silk suture after the surgery. (3) The mouse’s scalp was shaved and prepared for skin disinfection. Afterward, the scalp was removed to expose the skull. A specialized metal headpiece, as depicted in Fig. 6b, was affixed to the skull using dental cement. (4) The sham group underwent a minimally invasive incision on the mouse’s face area without any manipulation of the IoN. (5) The objective of attaching the head post to the mouse was to stabilize the head, which was conducted under temperature-controlled conditions of about 28 ℃. The mice were given sufficient food and water before and after the surgery, and all the procedures were carried out in a sterile environment. (6) Before imaging, position the infrared camera adjacent to the left face of the mouse such that the mouse’s face is away from the camera at a 90-degree angle, with a distance of around 5 cm. (7) The camera was activated to capture a 10 min video of the left facial region of the mouse. The recorded video was subjected to pre-processing steps identical to those detailed in the corresponding section 3.4. (8) To evaluate the facial pain threshold in the IoN-CCI mouse model, images of the facial region were captured on postoperative days 1, 8 and 15, followed by pain threshold measurement. The specific steps for the measurement are as follows: a series of von Frey filaments consisting of eight filaments (0.02 g, 0.04 g, 0.07 g, 0.16 g, 0.4 g, 0.6 g, 1.0 g and 1.4 g) are selected. Starting from the 0.02 g filament, the Up-and-Down method [14, 18, 19] is used to stimulate the mouse’s face. The filament should be bent into a “C” or “S” shape and applied for 6–8 seconds. The mouse’s withdrawal response is then observed and recorded: a negative response “O” is recorded if there is no response from the mouse, and a positive response “X” is recorded if there is a flinching or resistance response. In the event of a negative response, the next larger von Frey filament is used to stimulate the face. If there is a positive response, the next smaller filament is applied, with a few seconds between each stimulation. This process is repeated until “OX” or “XO” responses are observed. To obtain a sequence of responses consisting of combinations of “O” or “X”, four additional tests are conducted after the first “OX” or “XO” response. The filament size used in this sequence is recorded as the facial pain threshold of the mouse’s face. Subsequently, *t*-tests were employed to examine the significant differences between the sham and IoN-CCI groups on the first, eighth and fifteenth days. Additionally, the congruence of predictive outcomes regarding mouse pain intensity using TNPAN and measurements derived from the von Frey filaments was assessed for consistent trends of variation.

**Fig. 6.** The experimental protocol for the infraorbital nerve (IoN) constriction surgery. (a) The location of the IoN on the mouse was marked with a black arrow. (b) A metal head post was placed on the mouse’s head to immobilize it, as indicated by the red dashed box. (c) The site of the nerve constriction on the right side of the mouse’s face was identified and marked by the red dashed box.

## 4. Results and discussion

In this section, we initially constructed a pain grading dataset by combining an infraorbital nerve activity assay and a facial pain threshold assay. We utilized the objective pain grading dataset as the ground truth for model training, thereby overcoming the limitations associated with subjective assessments in prior investigations. Then, we investigated the performance of MAN, 3D-CNN and TNPAN, respectively. All the deep learning models are implemented in Python using PyTorch [26] as the backend. Finally, we validated the generalization capability of TNPAN using the IoN-CCI mouse model.

### 4.1 The results of pain grading

The accuracy and objectivity of pain grading labels play a crucial role in determining the performance of pain assessment models. Prior models have frequently relied on subjective evaluations of researchers as the supervisory signal during training, lacking objective criteria. In this section, we aim to construct a pain grading dataset that is both objective and accurate, derived from the outcomes of the infraorbital nerve activity assay and the mouse facial pain threshold assay (section 3.3).

The infraorbital nerve (IoN) is a crucial component of the trigeminal signaling pathway, being a significant branch of the trigeminal nerve. For an extended period, researchers [27, 28] have widely believed that the aberrant activation of the trigeminal nerve signaling pathway is frequently closely associated with trigeminal neuralgia. Thus, by scrutinizing the extent of abnormal IoN activity elicited by hypertonic saline stimulation, the pain state of the mouse model can be assessed. In this investigation, six distinct concentrations of NaCl solution (0.9%, 6%, 12%, 18%, 25% and 30%) were applied to the cornea of mice to induce aberrant excitation within the trigeminal nerve signaling pathway. The findings depicted in Fig. 7 demonstrate that the activity of IoN did not significantly differ from that of the control group when induced with 0.9% physiological saline (*t*-test, *p* = 0.263, n.s.). When using 6%, 12% and 18% NaCl solutions to stimulate the mice, a significant difference in the activity of IoN compared to the control group was observed (*p *< 0.01). However, one-way ANOVA (analysis of variance) results showed no significant difference among these three concentrations (*p* = 0.547, not significant). Moreover, in response to the stimulation with 25% and 30% NaCl solutions, the IoN demonstrated aberrant excitation, which not only exhibited significant differences when compared to the control group (*p *< 0.01) but also displayed higher levels of excitation than the 18% concentration (*p *< 0.01).

**Fig. 7.** The activity of the IoN was quantified by measuring the fluorescence intensity ratio Δ*F/F*. The error bars represent the standard error of the mean. No statistically significant difference was observed between the control group and the 0.9% saline group (*p* = 0.263, n.s). The significant differences among the 6%, 12% and 18% saline groups were determined by one-way ANOVA (*p* = 0.547, n.s). A significant difference was found between the 6% and 0.9% saline groups as determined by *t*-test (***p < 0.01). No significant difference was observed between the 25% and 30% saline groups using *t*-test (*p* = 0.106, n.s). However, the excitation levels were significantly higher in the 25% and 30% groups compared to the 18% group (***p < 0.01). IoN: infraorbital nerve; NaCl: sodium chloride.

It can be observed that the stimulation of the mouse cornea with six different concentrations of NaCl solution did not result in six distinct levels of excitation in the IoN. The relationship between the concentration of NaCl solution and the activity of IoN is not strictly linear, and it may exhibit a stair-step effect, as demonstrated in Fig. 7. Specifically, when the concentration of NaCl solution reaches a certain threshold, the excitation level of IoN may jump to the next level.

Next, we turned our attention to the facial pain threshold in TN mice. In 1965, Melzack and Wall [29] introduced the “gate control theory of pain”, which has since been expanded into various sub-theories. The fundamental concept of this theory is that nociceptors become more sensitive during painful conditions, leading to a lower pain threshold than normal. Consequently, measuring the facial pain threshold in mouse models can be used as an indirect measure of their pain status [30].

The experimental findings depicted in Fig. 8 indicate that the facial pain threshold of mice did not differ significantly between the 0.9% saline group and the control group (*p* = 0.799, n.s.). Stimulation with 6%, 12% and 18% saline solutions led to a significant reduction in the facial pain threshold of mice compared to the control group (*p *< 0.01). However, one-way ANOVA analysis did not reveal any significant difference among the three groups (*p* = 0.394, n.s.). Under the conditions of 25% and 30% saline solutions, the facial pain threshold of mice exhibited a significant decrease compared to 18%, indicating a stepwise effect between the concentration of saline solution and the intensity of pain (*p *< 0.01). However, no significant difference was found between the 25% and 30% groups (*p* = 0.742, n.s). These results support the existence of a staircase effect in the relationship between the concentration of saline solution and pain intensity.

**Fig. 8.** The facial pain thresholds of TN mouse model. The error bars represent the standard error of the mean. The facial pain threshold of TN mice did not show significant differences compared to the control group under the 0.9% saline condition (*p* = 0.799, n.s). However, under the 6%, 12% and 18% saline conditions, the facial pain thresholds of mice significantly decreased compared to 0.9% (**p < 0.05), with no significant difference observed among the three groups (*p* = 0.394, n.s). Moreover, under the 25% and 30% stimulation conditions, the facial pain thresholds of the mice further decreased compared to 18% (***p < 0.01), with no significant difference between the two groups (*p* = 0.742, n.s). These findings support the notion of a stepwise effect between the concentration of saline solution and the intensity of pain. NaCl: sodium chloride.

The above experimental results demonstrate that by using six different concentration gradients (0.9%, 6%, 12%, 18%, 25%, 30%) of hypertonic saline to stimulate the mouse cornea, acute trigeminal neuralgia of varying pain grades can be induced. This study classified the pain levels of the TN mouse model based on the degree of infraorbital nerve excitation and facial pain threshold. The pain was categorized into three levels: no pain (NP) for 0.9% saline, moderate pain (MP) for 6%, 12% and 18%, and high pain (HP) for 25% and 30% saline solutions. Then, we selected 3000 images from each pain level to construct a pain grading dataset, providing objective evidence for pain assessment. Each image is assigned a pain grade as the ground truth. During the training of the MAN, we enlisted the assistance of five experts to annotate the pupils and forelimbs of the mice for this pain grading dataset (as described in section 3.4). When training the models, the entire dataset was divided into 80% training dataset and 20% test datasets. All data are shown as mean ± SEM (standard error of the mean). Statistical analyses were done with Prism 8.1 (GraphPad, GraphPad Software LLC, San Diego, CA, USA). Comparisons were conducted with Student’s *t*-test to compare Gaussian distributions. Two-way ANOVA with repeated measures for multiple comparisons. When the *p* value < 0.05, the results were considered statistically significant.

### 4.2 Performance of motion awareness network (MAN)

The MAN was utilized to extract the dynamic behavioral characteristics of TN mice, which comprises two essential components: ResNet-50 and deconvolutional layers. The ResNet-50 is used as the feature detection module, which has been extensively trained on large-scale datasets such as ImageNet and has achieved excellent results in tasks such as object detection and human pose recognition [21]. Therefore, it is used to detect the positions of the mouse’s pupils and forelimb key points. In order to obtain the coordinate positions of the key points from MAN, eight deconvolutional layers [23], and eight readout layers were incorporated after the ResNet-50 module. Deconvolutional layers are used to up-sample the visual information and produce spatial probability densities.

The training process of the MAN is depicted in Fig. 9, and the loss gradually converged after around 500 k iterations. To evaluate the accuracy of the MAN, we generated a plot of the error distribution between the predicted coordinates of the eight key points by MAN and the ground truth coordinates (as demonstrated in Fig. 10). The errors were mainly concentrated within a 3-pixel range, indicating that the accuracy of MAN in recognizing the facial pupil and forelimb key points of mice is comparable to that of manual annotation. At present, the MAN based on ResNet-50 has attained a high level of accuracy in predicting the positions of the mouse’s pupil and forelimb key points, which satisfies the demands for extracting dynamic behavioral characteristics of the TN mouse.

**Fig. 9.** Corresponding RMSE between the human and the predicted results on training and test images (80%/20% splits). Human variability, as well as the 95% confidence interval, are depicted in black and gray. RMSE: root-mean-square error.

**Fig. 10.** Error comparison of eight body points between the model prediction and labels. The first row from left to right are limb1, limb2, limb3 and limb4, respectively. The second row from left to right is pupil1, pupil2, pupil3 and pupil4, respectively.

### 4.3 Performance of 3D convolutional neural network (3D-CNN)

The objective of this section is to evaluate the efficacy of various 3D-CNN architectures in extracting static texture characteristics of the face of the TN mouse model. The performance of the models will be evaluated based on accuracy, precision, recall, F1 and AUC. We aim to optimize the network structure of the 3D-CNN by adjusting its hyperparameters. We are aware that the size of the spatial convolutional kernel in 3D convolutional kernels determines the receptive field of the model. This, in turn, determines the model’s ability to capture information and plays a crucial role in extracting information and achieving effective feature representation. Thus, this study conducts a series of experiments to explore the optimal convolutional kernel structure by varying the size of the spatial convolutional kernel.

Table 3 shows the accuracy of 3D-CNN in pain assessment tasks with different spatial kernel sizes. The results demonstrate that the model attained the maximum accuracy when the spatial convolutional kernel size was set to 3 × 3. The findings indicate that larger spatial kernel sizes do not necessarily result in superior performance. As the size of the spatial kernel increases from 5 × 5 to 11 × 11, the accuracy of the model does not improve but instead decreases. This suggests that excessively large spatial kernels capture more coarse information, which can undermine the model’s capacity to make precise evaluations.

**Table 3.** Comparison of the 3D-CNN model’s accuracy (%) on the different kernel sizes with fixed temporal depth = 16.

Kernel Size	3 × 3	5 × 5	7 × 7	9 × 9	11 × 11
Accuracy (%)	82.84	77.73	76.98	74.60	72.46

In addition to the spatial kernel size, prior research [31, 32] has also highlighted that the temporal depth of 3D kernels can impact the model’s ability to represent spatiotemporal features and subsequently influence its inferential ability. Proper temporal depth is essential for capturing facial pain features in the TN mouse model. Therefore, this section also investigates the influence of different temporal depths of 3D convolutional kernels on the model’s accuracy. The results presented in Table 4 indicate that among the temporal depths examined (3, 5, 8, 16, 32 and 40), the 3D convolutional kernel with a temporal depth of 16 attained the highest accuracy for the model. This suggests that setting the time depth of the 3D convolutional kernel to 16 can efficiently capture the short-, mid- and long-range spatiotemporal features of TN mouse in the pain assessment task, leading to effective expression of static texture characteristics. However, excessively long time windows, such as those with a time depth of 32 or 40, can introduce additional irrelevant noise and thus decrease the model’s accuracy. Conversely, using too short a time window, such as one with a time depth of 3, 5 or 8, may not provide sufficient information for pain assessment.

**Table 4.** Comparison of the 3D-CNN model varying temporal depths with fixed kernel size = 3 × 3.

Temporal Depth	3	5	8	16	32	40
Accuracy (%)	63.63	77.91	78.87	82.84	78.31	76.46

According to the experimental findings presented in this section, the optimal configuration for the 3D-CNN was attained when the spatial kernel size was set to 3 × 3 and the temporal depth was set to 16, resulting in the highest accuracy for the pain assessment task. In the subsequent experiments, we maintain the same set of hyperparameters, including a spatial convolutional kernel size of 3 × 3 and a temporal depth of 16. The remaining training hyperparameters are set as follows: a batch size of 16, an initial learning rate of 0.0001, and a maximum of 100 epochs.

### 4.4 Performance of trigeminal neuralgia pain assessment network (TNPAN)

This section aims to evaluate the performance of the trigeminal neuralgia pain assessment network (TNPAN) and compare it with four baseline models. The architecture of TNPAN is described in section 3.5. The first baseline model, as described in paper [33], is the HOG-SVM (support vector machine) approach. This method involves extracting HOG features from the mouse’s face using the HOG algorithm, followed by utilizing SVM for pain assessment. The second baseline model, HOG-NN (neural network), is similar to the first one as it also extracts HOG features from the facial region of the mouse and utilizes a shallow neural network for pain assessment. The neural network comprises two hidden layers implemented with the ReLU function and an output layer implemented with the softmax function. The third baseline model proposed by Tuttle *et al*. [9] uses facial images of mice as input and employs a deep neural network to output the pain assessment results. The fourth baseline model proposed by Vidal *et al*. [10] comprises two modules, namely, an eye socket recognition module and a pain evaluation module.

To ensure consistency in the comparison results, all four baseline models were trained on the same dataset and hardware resources. The training process was repeated three times, and the evaluation metrics were calculated as the mean of the results from the three training runs. To further investigate the respective contributions of the two components (MAN and 3D-CNN) in TNPAN towards pain assessment, in addition to comparing with previous methods, a series of ablation experiments were conducted where only MAN or 3D-CNN was utilized for pain assessment. The dataset and training resources used in the experiments are consistent with those used in the previous experiments.

The results are presented in Table 5, which indicate that the proposed TNPAN obtains the highest accuracy of 92.27%, as well as the highest precision and recall, when compared to the four baseline models. The results suggest that TNPAN surpasses previous approaches in the pain assessment of TN mice. To explore the significance of dynamic behavioral characteristics of TN mice in pain assessment, this study compared the accuracy of utilizing only MAN with that of TNPAN. The results revealed that incorporating the fixed model reuse strategy to consider the dynamic behavioral characteristics of mice enhanced the accuracy of the model by 22.79%. These findings demonstrate that the dynamic behavioral characteristics of TN mice play a crucial role in pain assessment. Similarly, we investigated the role of the TN mouse’s static texture characteristics in pain assessment. The results showed that TNPAN achieved a 9.43% improvement in accuracy compared to using only 3D-CNN, indicating the importance of the static texture characteristics of the mouse’s face in pain assessment. Interestingly, the observation that the accuracy of using only 3D-CNN is 13.36% higher than using only MAN is noteworthy, as it suggests that the static texture characteristics of the mouse face are more influential than the dynamic behavioral characteristics in pain assessment. This could be attributed to the fact that mice engage in various non-pain-related behaviors, such as grooming, besides wiping their eyes when experiencing pain, which may result in misclassification by the model.

Overall, the pain assessment of TN mice can only be accurately performed by synergistically utilizing both dynamic behavioral characteristics and static texture characteristics.

**Table 5.** Comparison of the TNPAN (depth = 16, kernel size = 3 × 3) accuracy (%), precision (%), recall (%), F1 value (%), and area under the curve (AUC) with other baseline models.

No.	Model Structure	Accuracy (%)	Precision (%)	Recall (%)	F1 (%)	AUC
1	HOG-SVM	61.11	53.08	61.12	54.07	0.704
2	HOG-NN	72.48	62.76	72.10	65.43	0.792
3	Tuttle *et al*. [9]	75.48	65.76	75.36	68.78	0.817
4	Vidal *et al*. [10]	79.30	69.81	79.42	73.15	0.845
5	Only MAN	69.48	74.81	70.31	72.49	0.824
6	Only 3D-CNN	82.84	73.86	82.76	77.25	0.871
7	TNPAN	92.27	86.45	92.48	89.14	0.942

*MAN: motion awareness network; 3D-CNN: 3D convolutional neural network; TNPAN: trigeminal neuralgia pain assessment network; AUC: area under the curve; HOG-SVM: histogram of oriented gradients support vector machine; HOG-NN: histogram of oriented gradients neural network.*

### 4.5 The performance of TNPAN in the IoN-CCI mouse model

In the field of machine learning, a model’s practicality is determined by its generalization ability. To evaluate the generalization ability of TNPAN, this study conducted a validation on the IoN-CCI mouse model. The results, as illustrated in Fig. 11, exhibit a persistent decline in facial pain threshold over time in the IoN-CCI mouse model. On postoperative days 8 and 15, a significant difference in facial pain threshold was observed between the IoN-CCI mouse model and the sham-operated group (*p *< 0.01). These findings suggest that facial pain intensity in the mice progressively increased, possibly due to the inflammatory response in the facial region of the mice [11]. Next, TNPAN was applied to the IoN-CCI mouse model, and it was found that during the initial stage of modeling, the predictions of TNPAN for the IoN-CCI mice were in agreement with those of the sham group. As time progressed, the results of TNPAN on the IoN-CCI mice continued to improve, reaching a level of moderate pain on day 8 and high pain on day 15. The consistency between the predictive results of TNPAN and the changing trend of facial pain threshold in the IoN-CCI mouse model suggests the feasibility of TNPAN application for pain assessment in this model, as well as the generalization ability of TNPAN. Clinically, orofacial pain encompasses various conditions including orofacial pain attributed to disorders of dentoalveolar and anatomically related structures such as dental pain, gingival pain and myofascial orofacial pain, including primary myofascial orofacial pain and acute primary myofascial orofacial pain. The dual-channel feature fusion strategy proposed in this study not only accounts for static structural features but also encompasses dynamic behavioral features, which are relevant in pain conditions beyond neuropathic pain. Hence, the potential applicability of TNPAN extends to other orofacial pain disorders.

**Fig. 11.** The performance of TNPAN in the IoN-CCI mouse model. (a) Changes in facial pain threshold in the IoN-CCI mouse model. (b) The predictive results of TNPAN for pain intensity at different time points in the IoN-CCI mouse model, with NP indicating no pain, MP indicating moderate pain, and HP indicating high pain. *p < 0.05; **p < 0.01; ***p < 0.001; n.s, not significant. IoN: infraorbital nerve; TNPAN: trigeminal neuralgia pain assessment network.

## 5. Conclusions

This study presents a non-contact trigeminal neuralgia pain assessment network (TNPAN) based on dual-channel feature fusion to mitigate the aforementioned limitations. Firstly, we constructed an objective pain grading dataset, which remedied the limitations of prior studies that relied on subjective evaluation as supervisory signals. Subsequently, a dual-channel fusion strategy was utilized to extract both static texture characteristics (3D-CNN) and dynamic behavioral characteristics (MAN) from the facial region of mice, resulting in a substantial improvement in the accuracy of pain assessment (92.27%). In contrast to the deep learning-based models proposed by Vidal and Neff [34, 35] and 3D-CNNs with fixed and uniform kernel depths [36, 37], this study examined the performance of 3D-CNNs with diverse hyperparameters to identify the optimal network architecture. The results indicated that the 3D-CNN could effectively capture the static texture characteristics of the mouse face by configuring the spatial size of the 3D convolution kernel to 3 × 3 and the temporal window size to 16. The results of the ablation experiments demonstrated that solely considering the dynamic functional features (MAN) or the static structural features (3D-CNN) of TN mice did not yield satisfactory accuracy levels (69.48% and 82.84%, respectively). A more accurate assessment of pain intensity could only be achieved by comprehensively integrating both the static texture and dynamic behavioral characteristics of the TN mouse face.

To further explore the generalizability of TNPAN in pain assessment, this study conducted experiments on the mouse model of infraorbital nerve chronic constriction injury (IoN-CCI). The prediction outcomes of TNPAN demonstrated that the pain intensity of mice was initially low after conducting the IoN-CCI operation, and it gradually intensified over time. On the 15th day after the infraorbital nerve chronic constriction injury surgery, the pain intensity reached its maximum level. TNPAN’s predictions were consistent with the facial pain threshold of mice determined by the von Frey filament test, thus confirming the generalizability of TNPAN for pain assessment.

The dual-channel feature fusion strategy for pain assessment proposed in this study, which integrates static texture and dynamic behavioral characteristics, has substantial implications for the advancement of pain assessment in the field. Furthermore, exploring the feasibility of applying this pain assessment strategy to other species, such as primates, including monkeys, represents a promising avenue for future investigation.
